# The effect of medical insurance on catastrophic health expenditure: evidence from China

**DOI:** 10.1186/s12962-020-00206-y

**Published:** 2020-02-27

**Authors:** Jian Sun, Shoujun Lyu

**Affiliations:** 1 School of International and Public Affairs, Shanghai Jiao Tong University, No. 1954 Huashan Road, Xuhui District, Shanghai, 200030 China; 2 China Institute for Urban Governance, Shanghai Jiao Tong University, No. 1954 Huashan Road, Xuhui District, Shanghai, 200030 China

**Keywords:** Medical insurance, Catastrophic health expenditure, Concentration index, Random effects panel Probit regression model, China

## Abstract

**Background:**

The Chinese government has established a nationwide multiple-level medical insurance system. However, catastrophic health expenditure (CHE) causes great harm to the quality of life of households and pushes them into poverty. The objective of this paper is to assess the effect of medical insurance on CHE in China and compare the financial protection effects of different medical insurances.

**Methods:**

Panel data were obtained from China Family Panel Studies (CFPS) conducted in the years of 2012, 2014, and 2016. CHE incidence was measured by performing a headcount, and its intensity was estimated using overshoot and mean positive overshoot (MPO). In addition, concentration index (CI) was used to measure the degree of socioeconomic inequality of CHE occurrence. Furthermore, random effects panel Probit regression model was employed to assess the effect of medical insurance on CHE. Lastly, random effects panel Logit regression model was adopted to perform a robustness check.

**Results:**

From 2012 to 2016, the total CHE incidence jumped from 15.05% to 15.24%, and the CI in CHE changed from − 0.0076 to − 0.1512. Moreover, the total overshoot increased from 0.0333 to 0.0344, while the total MPO grew from 0.2213 to 0.2257. Furthermore, the global regression results show that residents covered by Supplementary Medical Insurance (SMI) were linked to a decreased probability of experiencing CHE. In addition, the regression results by gender indicate that SMI coverage for male residents had a significant effect on the prevention of CHE, while the effect was not significant for female residents. The regression results by health status show that SMI had a significant impact on reducing the likelihood of CHE occurrence for healthy residents, whilst the impact was not significant for unhealthy residents. Lastly, the robustness check results were consistent with those of previous findings.

**Conclusion:**

The results of this study suggest that CHE incidence and intensity became relatively higher among households. In addition, CHE occurrence was concentrated among the poorer households and the equality status worsened. Moreover, financial protection effects of the four medical insurance schemes against CHE varied significantly. Furthermore, the protection effect of SMI against CHE shows significant gender and health status disparities.

## Background

Universal health coverage (UHC) is the goal of health policy makers, and medical insurance is the recommended strategy to achieve that goal [1]. The Chinese government has established a nationwide multiple-level medical insurance system. The main purpose of the medical insurance system is to improve the availability of health care services, effectively lower health care expenses, and reduce the incidence of catastrophic health expenditure. National basic medical insurance schemes are currently implemented in China, covering more than 1.35 billion residents. Moreover, the insured rate reached more than 95% and the reimbursement level rose steadily in both urban and rural areas, therefore a universal medical insurance system has basically formed [2, 3]. National basic health insurance schemes attach more importance to the financial protection in the case of critical illness, and it consists of three key programs: Urban Employee Basic Medical Insurance (UEBMI) designed for the employed urban residents, established in 1998; Urban Resident Basic Medical Insurance (URBMI) designed for urban residents without a formal job, initiated in 2007; and New Rural Cooperative Medical Insurance (NRCMI) designed for rural residents, launched in 2003. Supplementary Medical Insurance (SMI) provided by commercial insurance agencies or industry associations serves as an important supplementary insurance to national basic health insurance schemes, and its aim is to meet residents’ multi-level demands for health care, which mainly consists of commercial medical insurance, employee medical subsidy for large medical expenses, enterprise supplementary medical subsidy, public servant medical subsidy, and employee mutual medical insurance, targeting mainly the upper class [4]. In addition, SMI has relatively higher premiums and reimbursement rates than other insurances. The proportion of out-of-pocket (OOP) payments in total health expenditures shows a significant reduction after the introduction of medical insurance. However, there exist great disparities in terms of financial protection level among different medical insurances. For example, UEBMI has premiums 10 times higher than either URBMI or NRCMI [5]. Additionally, fragmentation in the medical insurance system increases the cost of financing and management, and impairs its efficiency and sustainability.

In recent years, the Chinese government has released several policy documents concerning the development of medical insurance. In January 2016, Opinions on Integrating the Basic Medical Insurance System for both Urban and Rural Residents was issued by the State Council of China. The policy document clarified that it is necessary to integrate URBMI and NRCMI in order to establish a unified system of basic medical insurance schemes for both urban and rural residents by unifying coverage, financing policy, reimbursement level, security directory, the management of appointed medical institutions, and the management of medical insurance funds. In October 2016, the Chinese government announced the “Healthy China 2030” blueprint, the goal of which is to provide universal health security for all citizens by 2030 [6]. In October 2017, General Secretary Xi Jinping stated at the 19th National Congress of the Communist Party of China that it is imperative to strengthen the social security system and improve the unified system of basic medical insurance schemes for both urban and rural residents.

Catastrophic health expenditure (CHE) is defined as a general term used to describe all types of health expenditures that pose a threat to the financial capacity of a household to maintain its subsistence needs, which contributes to the inequality of health financing, causes great harm to the quality of life of households and pushes them into poverty [7, 8]. In general, CHE often occurs to the poor due to their financial condition. Considering the serious consequences of CHE and the principle of health financing, it is widely accepted that the objective of health policy is to protect residents against CHE [9–11]. The World Health Organization (WHO) proposed that CHE occurs when health care payments equal or exceed 40% of a household’s non-subsistence income [12]. Indeed, residents in China are faced with higher economic burden of diseases, and the overall incidence of CHE is about 13%, while the incidence of CHE can be more than 50% for low-income rural families [13, 14].

Previous research on the impact of medical insurance on disease economic risk mainly focused on the effect of medical insurance on CHE. Most studies considered that medical insurance, serving as a type of health risk dispersed mechanism, could decrease OOP health expenditures to some extent, thus reducing the probability of CHE occurrence. Zhou et al. found that NRCMI could play only a limited role in protecting households from CHE due to tuberculosis care [15]. Yardim and Cilingiroglu discovered that more people in Turkey benefited from health insurance by 2006 and were better protected from catastrophic medical expenses than in many other countries [16]. Likewise, a study in India indicated that community-based health insurance could protect poor households against catastrophic expenditures [17]. In addition, Aryeetey et al. provided evidence that enrolment into the National Health Insurance Scheme in Ghana reduced OOP expenditure, and provided financial protection against catastrophic expenditures and reduced poverty [18].

Medical insurance, as a type of price subsidy, can reduce residents’ actual costs of seeking health care and increase their demands for health care, while it may not necessarily reduce the possibility of experiencing CHE. A study conducted by Wagstaff and Lindelow suggested that people with medical insurance in China tended to seek health care services in higher-level medical institutions more frequently than the uninsured did, which increased the risk of high and catastrophic spending [19]. Li et al. also reported that households covered by NRCMI had similar levels of CHE as those without health insurance, so NRCMI failed to prevent CHE [20]. Similarly, a study by Chen et al. suggested that NRCMI had no significant impact on the incidence of CHE, and income and health status were the main influence factors of CHE for rural residents [21]. Meng et al. concluded that remarkable increases in medical insurance coverage and inpatient reimbursement had not been accompanied by reductions in CHE [22].

With the depth development of health care system reform in China, medical insurance is confronted with challenges as well as opportunities. In the past few decades, the medical insurance system has been designed to protect against CHE by reducing OOP health expenditures, whereas the protective effect has remained unclear. Moreover, little research has been published to compare the financial protection effects of different medical insurances. The objective of this paper is to assess the effect of medical insurance on CHE in China and compare the financial protection effects of the different medical insurances using panel data. The results of this study will shed light on policy suggestions regarding how to optimize medical insurance policy to reduce CHE incidence and intensity.

## Methods

### Data sources and statistical analysis

Considering that panel data regression can control the impacts of omitted variables on econometric results, we obtained data from China Family Panel Studies (CFPS) conducted in the years of 2012, 2014, and 2016 to constitute balanced panel data. CFPS is performed by Institute of Social Science Survey (ISSS), Peking University, China. It reflects the transition of society, economics, population, education, health and so forth in China, and provides high-quality micro-data for both public policy analysis and academic research. Moreover, CFPS is a nationally representative, large-scale, and multidisciplinary social tracking survey project, covering 25 provinces in China, and respondents included in this survey all aged 16 and above. We removed the missing and abnormal values from the CFPS databases. Furthermore, according to the household ID, we matched the households in the three waves survey to ensure the accuracy and authenticity of the panel data. Finally, 5768 valid samples from the three waves survey, including 17,304 observations, were included in this study.

STATA software version 15.0 SE was used to conduct descriptive statistics, calculate headcount, overshoot, mean positive overshoot, and construct random effects panel Probit regression model and random effects panel Logit regression model. All tests were two-sided and a *p*-value < 0.05 indicates statistical significance. In addition, Microsoft Excel version 2013 was employed to draw figures.

### Variables

Description of variables is listed in Table 1. The dependent variable, that is CHE, is a dummy variable indicating whether CHE occurs. CHE equals 1 if OOP health expenditures as a proportion of its non-food household expenditure equals or exceeds 0.4, and zero otherwise.

**Table 1:** Description of variables

Variable	Variable description
CHE	Incurring CHE = 1, without CHE = 0
UEBMI	Covered by UEBMI = 1, without UEBMI = 0
URBMI	Covered by URBMI = 1, without URBMI = 0
NRCMI	Covered by NRCMI = 1, without NRCMI = 0
SMI	Covered by SMI = 1, without SMI = 0
Marital status	Married = 1, divorce or other = 0
Gender	Male = 1, female = 0
Hukou status^a^	Urban hukou = 1, rural hukou = 0
Have chronic diseases	Yes = 1, no = 0
Health status	Unhealthy = 1, healthy = 0

^a^There are two main types of hukou in China, an urban type and a rural type; this classification is according to a person’s birthplace

With reference to previous studies [23, 24], medical insurance variables were adopted as independent variables, while demographic variables and health variables were chosen as control variables in this study. Firstly, medical insurance variables included four dummy variables: UEBMI, URBMI, NRCMI, and SMI, indicating whether they were covered by the medical insurance. Secondly, demographic variables included three dummy variables: marital status, gender, and hukou status. Thirdly, health variables included two dummy variables: have chronic diseases and health status.

### Measuring CHE incidence and intensity

CHE incidence was measured by conducting a headcount. Headcount refers to the percentage of households whose OOP health expenditures as a proportion of expenditure equals or exceeds a threshold. While there is no consensus on the threshold, in line with previous studies [23, 25], we set the threshold at 40% of a household’s non-food expenditure in a year. Headcount was measured as follows:

$$Headcount = \frac{1}{N}\sum\limits_{i = 1}^{N} {E_{i} }$$

where *N* represents the sample size, *E*_*i*_ equals 1 if OOP health expenditures for household *i* as a proportion of its non-food expenditure equals or exceeds the threshold and zero otherwise.

CHE intensity was estimated using overshoot and mean positive overshoot (MPO). Overshoot is the average amount by which payments, as a proportion of expenditure, exceed the threshold. MPO suggests the degree by which the average OOP health expenditures of a household exceed the threshold [26].

Overshoot and MPO were calculated as follows:

$$Overshoot = \frac{ 1}{N}\sum\limits_{i = 1}^{N} {E_{i} } \, \left( {\frac{{M_{i} }}{{x_{i} - f_{i} }} - z} \right)$$

where *N* is the sample size, *M*_*i*_ refers to OOP health expenditures, *x*_*i*_ denotes total consumption expenditure, *f*_*i*_ stands for the food expenditure, and *z* is equal to 40% [27].

$$MPO = \frac{O}{H}$$

where *O* is overshoot, and *H* refers to headcount.

### Concentration index

Concentration index (CI) has long been extensively applied in the literature to assess inequality in health care [12]. In this study, CI was used to measure the degree of socioeconomic inequality of CHE occurrence. CI is defined as twice the area between the concentration curve and the line of equality [28]. Moreover, CI is sensitive to the demographic changes among different socioeconomic groups and reflects the effect of socioeconomic status on inequality in health care, ranging between − 1 and + 1; the greater the absolute value of CI indicates a greater degree of inequality [29, 30]. A value of 0 indicates absolute equality of CHE occurrence; a negative value indicates a concentration of CHE occurrence among poorer households; a positive value indicates a concentration of CHE occurrence among richer households. The formula adopted for computing CI is:

$$C = \frac{2}{\mu }cov\left( {x_{i} } \right.,\left. {r_{i} } \right)$$

where *C* denotes the CI, *μ* indicates the mean of CHE indicator, *x*_*i*_ is CHE indicator, and *r*_*i*_ is the fractional rank of annual income.

### Random effects panel regression model

Considering the fact that the dependent variable is a dummy variable, it not suitable to use OLS regression model. In accordance to previous studies [31, 32], Probit regression model is suitable in this circumstance. Generally, panel Probit regression model is divided into random effects panel Probit regression model and fixed effects panel Probit regression model. Moreover, the fixed effects panel Probit regression model has stricter data requirements, and dummy variables used in this study, such as UEBMI, URBMI, and NRCMI, may be omitted as fixed effects; the fixed effects panel Probit regression model cannot include variables that remain unchanged over time, since the gender variable used in this study remains unchanged over time, we employed random effects panel Probit regression model to assess the effect of medical insurance on CHE based on the panel data of CFPS. The empirical Probit regression model is as follows:

$$Prob\left( {y_{it} } \right) = \beta_{0} + \beta_{1} *Insurance_{it} + \beta_{2} *Demographics_{it} + \beta_{3} *Health_{it} + \varepsilon_{it}$$

where *y*_*it*_ denotes the probability of CHE occurrence for resident *i* in the period *t*, *β*_*0*_ is the intercept term, *β*_*1*_, *β*_*2*_, and *β*_*3*_ are all the regression coefficients, *Insurance*_*it*_ indicates the medical insurance variables, *Demographics*_*it*_ is a vector of demographic variables and *Health*_*it*_ is a vector of health variables, and *ε*_*it*_ is the error term.

To make the regression results more convincing, random effects panel Logit regression model was adopted to perform a robustness check, and the Logit regression model is as follows:

$$\log \left( {P_{it} } \right) = \ln \left( {\frac{{P_{it} }}{{ 1- P_{it} }}} \right) = \beta_{0} + \beta_{1} *Insurance_{it} + \beta_{2} *Demographics_{it} + \beta_{3} *Health_{it} + \varepsilon_{it}$$

where *P*_*it*_ is the likelihood of incurring CHE for resident *i* in the period *t*, and *P*_*it*_/(1 − *P*_*it*_) is the odds ratio (OR) for CHE occurrence.

## Results

### Descriptive statistics

Socio-demographic characteristics of the respondents in 2012, 2014, and 2016 are summarized in Table 2. In 2016, 879 households suffered from CHE, and 49.19% of respondents were male. Moreover, 73.79% of them lived in rural area. From 2012 to 2016, the percentage of respondents being married dropped from 86.43% to 85.33%. At the same time, more than 60% of the respondents were healthy. In addition, the percentage of them having chronic diseases increased from 14.44% to 20.93%. In the year of 2016, the number of respondents covered by UEBMI, URBMI, NRCMI, and SMI were 941, 426, 4296, and 66, respectively.

**Table 2:** Socio-demographic characteristics of the respondents

Characteristic	2012		2014		2016	
	N	Percent (%)	N	Percent (%)	N	Percent (%)
CHE						
Incurring CHE	868	15.05	809	14.03	879	15.24
Without CHE	4900	84.95	4959	85.97	4889	84.76
Gender						
Male	2837	49.19	2837	49.19	2837	49.19
Female	2931	50.81	2931	50.81	2931	50.81
Marital status						
Married	4985	86.43	4975	86.25	4922	85.33
Divorce or else	783	13.57	793	13.75	846	14.67
Hukou status						
Urban hukou	1456	25.24	1474	25.55	1512	26.21
Rural hukou	4312	74.76	4294	74.45	4256	73.79
Have chronic diseases						
Yes	833	14.44	1150	19.94	1207	20.93
No	4935	85.56	4618	80.06	4561	79.07
Health status						
Unhealthy	2277	39.48	1980	34.33	2246	38.94
Healthy	3491	60.52	3788	65.67	3522	61.06
UEBMI						
Covered by UEBMI	822	14.25	874	15.15	941	16.31
Without UEBMI	4946	85.75	4894	84.85	4827	83.69
URBMI						
Covered by URBMI	380	6.59	428	7.42	426	7.39
Without URBMI	5388	93.41	5340	92.58	5342	92.61
NRCMI						
Covered by NRCMI	4375	75.85	4325	74.98	4296	74.48
Without NRCMI	1393	24.15	1443	25.02	1472	25.52
SMI						
Covered by SMI	36	0.62	65	1.13	66	1.14
Without SMI	5732	99.38	5703	98.87	5702	98.86

### CHE incidence in households

**Fig. 1:**
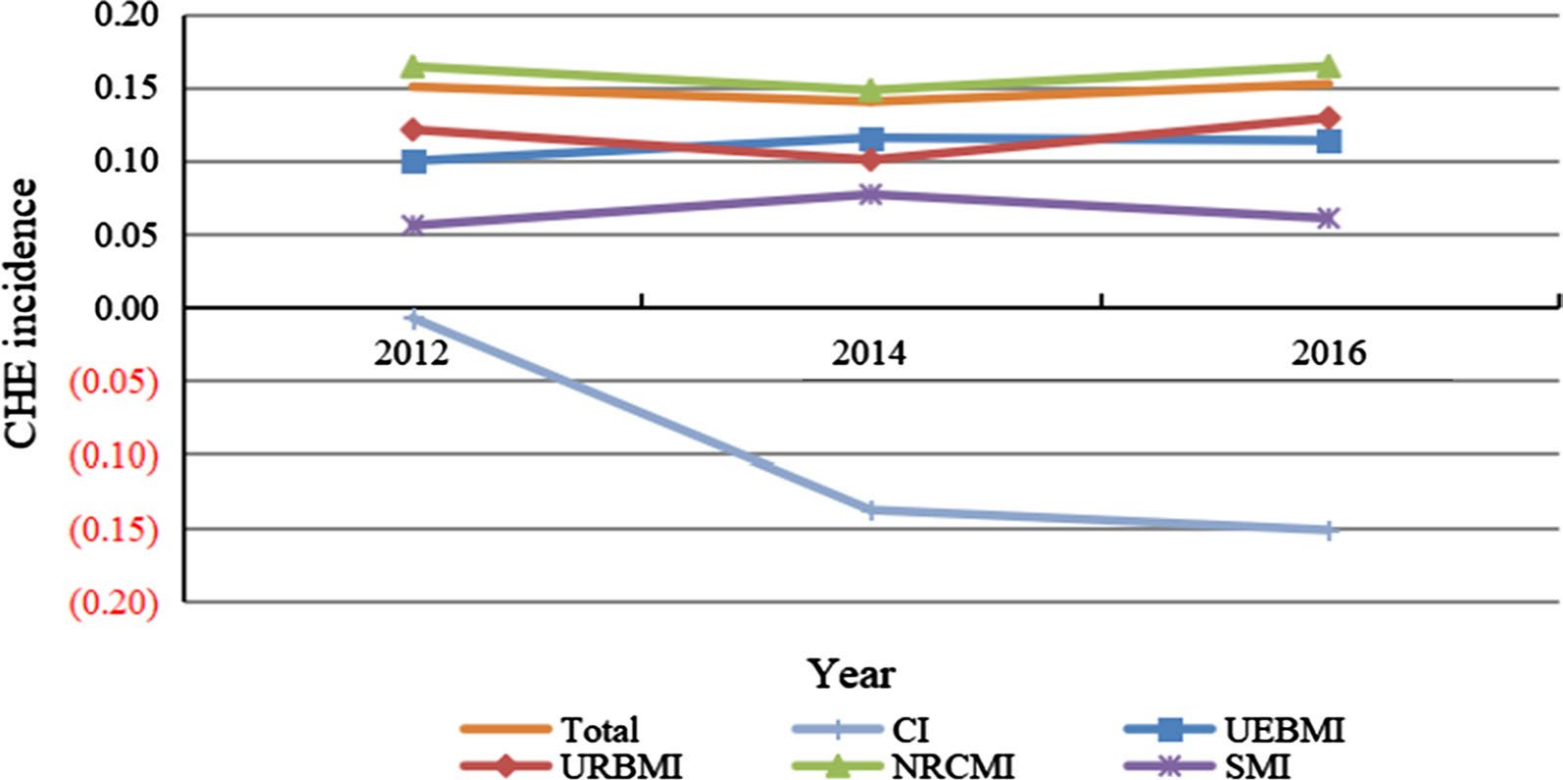
Trends in CHE incidence of households from 2012 to 2016

Figure 1 displays the trends in CHE incidence of households from 2012 to 2016. Overall, the total CHE incidence jumped from 15.05% in 2012 to 15.24% in 2016. It should be noted that during the study period the CI in CHE substantially changed from − 0.0076 to − 0.1512, implying that the equality status worsened. CHE incidences in households insured by the four medical insurances all showed overall upward trends. Among the four insurances, CHE incidence of households with SMI was the lowest during study period, which implies that its financial protection effect was the best. On the contrary, CHE incidence of households covered by NRCMI was the highest, which indicates that its protection effect was the worst.

### CHE intensity in households

**Fig. 2:**
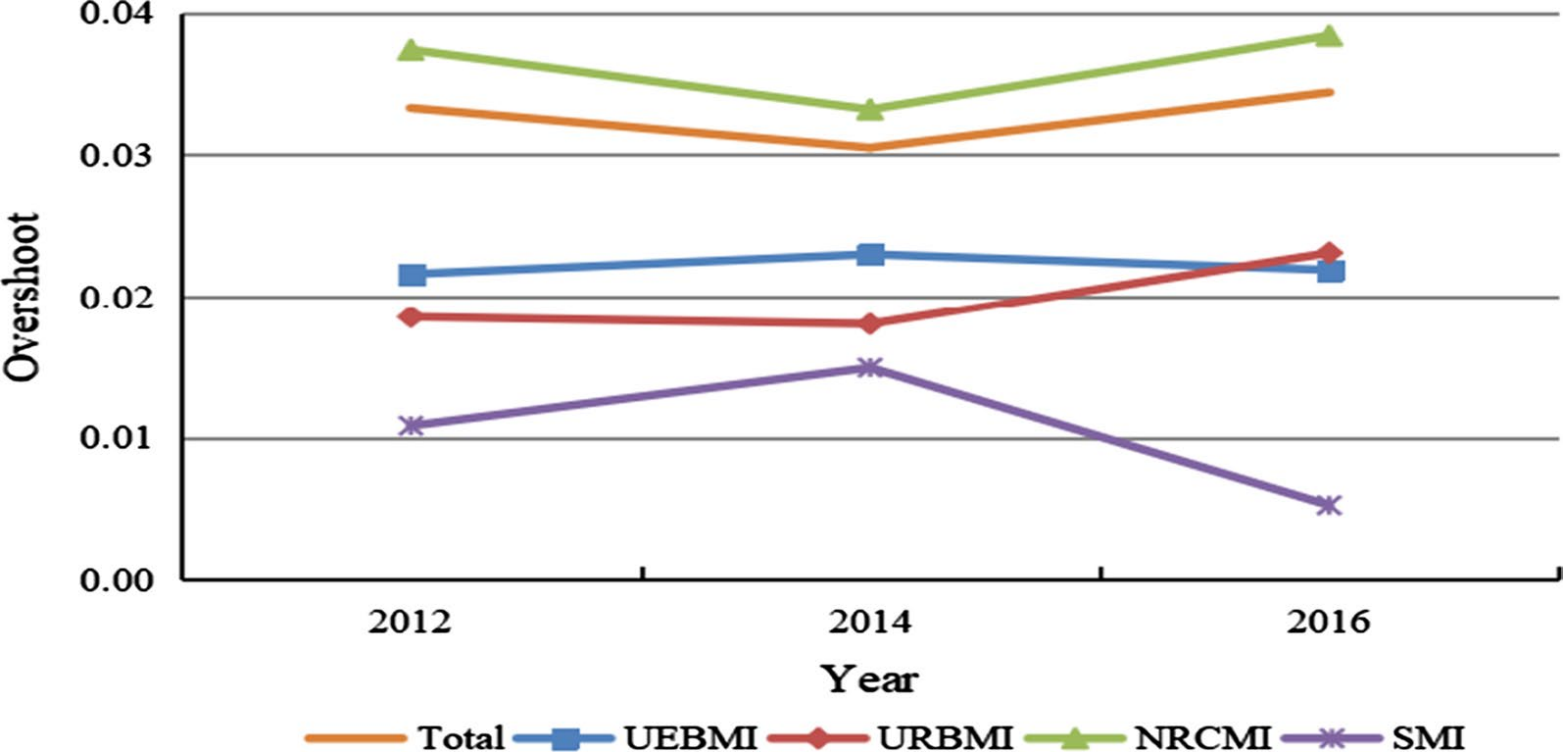
Trends in overshoot of households from 2012 to 2016

Figure 2 presents the trends in overshoot of households from 2012 to 2016. From 2012 to 2016, the total overshoot slightly increased from 0.0333 to 0.0344. Overshoots of households insured by UEBMI, URBMI, and NRCMI were all increased, while overshoots of households insured by SMI exhibited an overall downward trend. During the same period, overshoots of households covered by NRCMI, URBMI, as well as UEBMI were much higher than those covered by SMI, indicating that the financial protection level of basic medical insurance schemes should be further promoted.

**Fig. 3:**
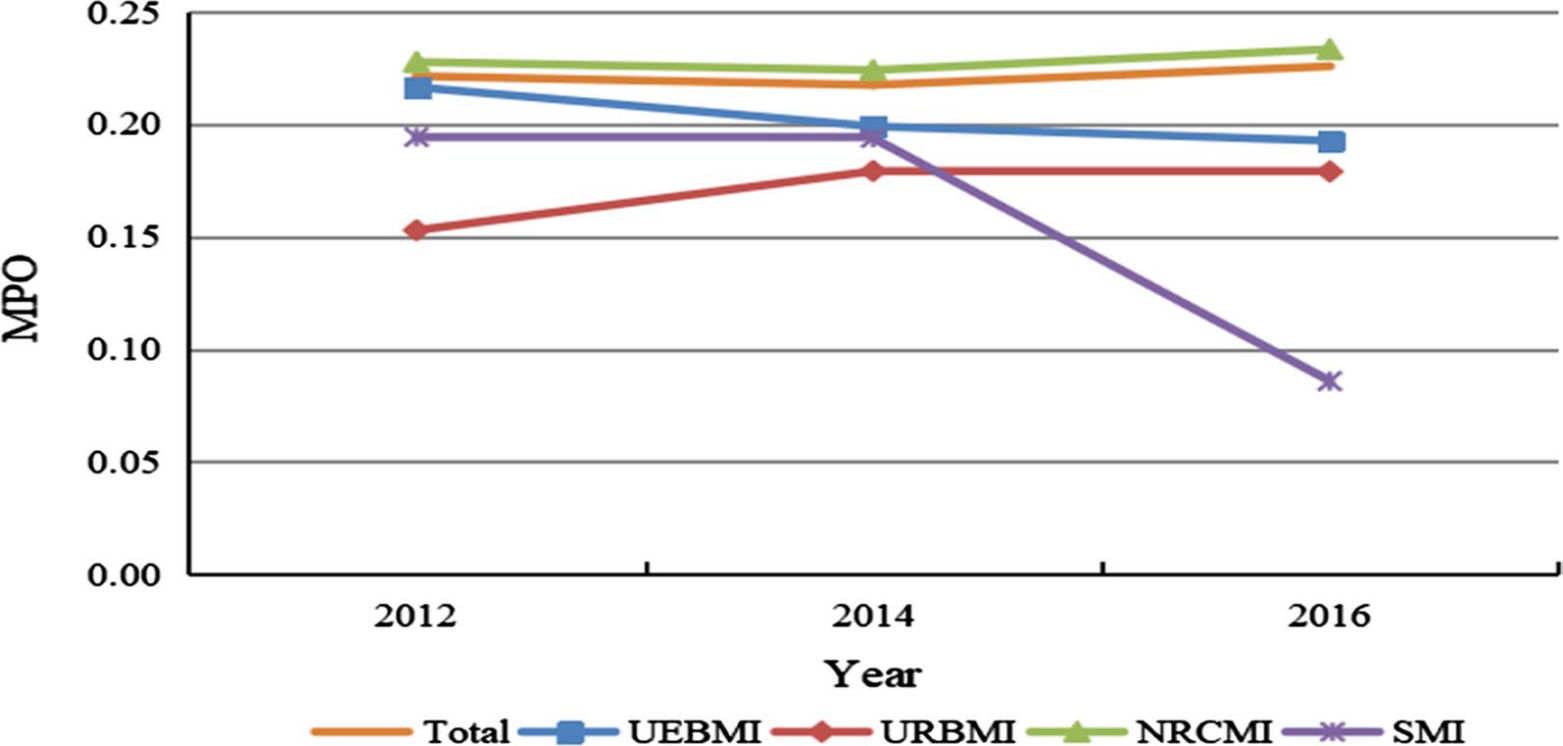
Trends in MPO of households from 2012 to 2016

Figure 3 visualizes the trends in MPO of households from 2012 to 2016. Total MPO decreased from 0.2213 in 2012 to 0.2174 in 2014, and then grew to 0.2257 in 2016. MPO of households insured by URBMI and NRCMI rose by 17.08% and 2.50%, respectively. In contrast, MPO of households covered by UEBMI and SMI dropped by 11.00% and 55.82%, respectively. Among the medical insurances, MPO of households with NRCMI was the highest in the year of 2016, while MPO of households covered by SMI was the lowest.

### Effect of medical insurance on CHE

#### Global regression results

Table 3 empirically examines the impact of medical insurance on CHE. The results show that residents covered by SMI were linked to a decreased probability of experiencing CHE compared to those without SMI (coefficient = − 0.425, *P* < 0.05). Furthermore, residents insured by UEBMI, URBMI, and NRCMI had no statistically significant association with lower probability of incurring CHE. Moreover, it was found that being unmarried, unhealthy, and having chronic diseases were significantly associated with an increase in the risk of CHE. Nevertheless, gender and hukou status had no statistically significant correlation with the occurrence of CHE.

**Table 3:** Results of global regression analysis

Variable	Coef.	Std. err.
Gender	0.024	0.035
Marital status	− 0.169	0.046**
Hukou status	− 0.038	0.070
Have chronic diseases	0.441	0.036**
Health status	0.465	0.032**
UEBMI	− 0.077	0.086
URBMI	− 0.093	0.097
NRCMI	0.177	0.098
SMI	− 0.425	0.194*
Constant	− 1.587	0.111**

* *p* < 0.05, ** *p* < 0.01

#### Regression results by gender

Table 4 investigates the effect of medical insurance on CHE by gender. The regression results indicate that SMI coverage for male residents had a significant effect on the prevention of CHE (coefficient = − 0.879, *P* < 0.05), while the effect was not significant for female residents (coefficient = − 0.147, *P* > 0.05). In addition, UEBMI, URBMI, and NRCMI coverage for both male and female residents was not linked to a lower probability of suffering from CHE. Moreover, being unmarried, unhealthy, and having chronic diseases were important factors that influence the occurrence of CHE for both male and female residents. However, hukou status had no statistically significant association with the probability of CHE occurrence for both male and female residents.

**Table 4:** Regression results by gender

Variable	Male		Female	
	Coef.	Std. err.	Coef.	Std. err.
Marital status	− 0.162	0.065*	− 0.184	0.066**
Hukou status	− 0.005	0.097	− 0.072	0.102
Have chronic diseases	0.486	0.053**	0.400	0.050**
Health status	0.489	0.046**	0.439	0.045**
UEBMI	− 0.096	0.110	− 0.030	0.139
URBMI	− 0.156	0.133	0.010	0.147
NRCMI	0.133	0.131	0.262	0.151
SMI	− 0.879	0.370*	− 0.147	0.243
Constant	− 1.533	0.145**	− 1.646	0.168**

* *p* < 0.05, ** *p* < 0.01

#### Regression results by health status

Table 5 explores the impact of medical insurance on CHE by health status. The results show that SMI had a significant impact on reducing the likelihood of CHE occurrence for healthy residents (coefficient = − 0.595, *P* < 0.05), whilst the impact was not significant for unhealthy residents (coefficient = − 0.212, *P* > 0.05). Moreover, unhealthy residents who got married could reduce the odds of experiencing CHE (coefficient = − 0.265, *P* < 0.01). However, marital status was not correlated with the probability of incurring CHE for healthy residents (coefficient = − 0.105, *P* > 0.05). Furthermore, having chronic diseases was related to an increase in CHE probability for both healthy and unhealthy residents.

**Table 5:** Regression results by health status

Variable	Healthy residents		Unhealthy residents	
	Coef.	Std. err.	Coef.	Std. err.
Gender	0.002	0.047	0.053	0.049
Marital status	− 0.105	0.063	− 0.265	0.067**
Hukou status	− 0.081	0.092	− 0.013	0.110
Have chronic diseases	0.453	0.062**	0.445	0.046**
UEBMI	− 0.076	0.113	− 0.098	0.137
URBMI	− 0.092	0.133	− 0.130	0.146
NRCMI	0.137	0.128	0.183	0.156
SMI	− 0.595	0.288*	− 0.212	0.286
Constant	− 1.637	0.148**	− 1.045	0.171**

* *p* < 0.05, ** *p* < 0.01

#### Robustness check

Robustness check results are shown in Table 6. The global regression results show that SMI was related to a decrease in CHE occurrence (coefficient = − 0.811, *P* < 0.05). Besides, the regression results by gender and health status, respectively indicate that SMI had a significant effect on reducing the likelihood of CHE occurrence for both male and healthy residents, while the effect was not significant for female and unhealthy residents. Additionally, residents covered by UEBMI, URBMI, and NRCMI had no significant association with the decrease in probability of experiencing CHE. These results were consistent with those generated by random effects panel Probit regression models and provided positive support to our results. As a result, our regression results were highly robust for further analysis.

**Table 6:** Robustness check results

Variable	Global regression	Regression by gender		Regression by health status	
		Male	Female	Healthy residents	Unhealthy residents
Gender	0.044 (0.063)			− 0.000 (0.087)	0.090 (0.086)
Marital status	− 0.303 (0.083)**	− 0.293 (0.117)*	− 0.329 (0.118)**	− 0.192 (0.117)	− 0.459 (0.116)**
Hukou status	− 0.074 (0.127)	− 0.018 (0.176)	− 0.131 (0.185)	− 0.159 (0.171)	− 0.021 (0.192)
Have chronic diseases	0.781 (0.064)**	0.866 (0.094)**	0.707 (0.088)**	0.827 (0.112)**	0.774 (0.079)**
Health status	0.839 (0.058)**	0.883 (0.082)**	0.789 (0.081)**		
UEBMI	− 0.132 (0.157)	− 0.160 (0.202)	− 0.054 (0.252)	− 0.147 (0.213)	− 0.167 (0.238)
URBMI	− 0.169 (0.177)	− 0.276 (0.245)	0.013 (0.267)	− 0.179 (0.250)	− 0.224 (0.255)
NRCMI	0.314 (0.179)	0.238 (0.238)	0.467 (0.274)	0.237 (0.239)	0.326 (0.272)
SMI	− 0.811 (0.371)*	− 1.743 (0.770)*	− 0.287 (0.449)	− 1.132 (0.570)*	− 0.403 (0.515)
Constant	− 2.781 (0.202)	− 2.686 (0.264)	− 2.886 (0.304)	− 2.893 (0.276)	− 1.795 (0.299)

* *p* < 0.05, ** *p* < 0.01

## Discussion

Using CFPS panel data to explore the effect of medical insurance on CHE, this study suggests that total CHE incidence, overshoot, and MPO exhibited overall rising trends from 2012 to 2016, which implies that CHE incidence and intensity became relatively higher among households. In addition, CHE incidence in China was higher than that in some other developing countries, such as Brazil, India, Russia, et al. [33] Possible explanations could be divided into four aspects. Firstly, a family planning policy has been implemented in China for more than three decades, thus the household size is shrunken, reducing the ability to deal with catastrophic health payments. Secondly, health services utilization, which is an important factor resulting in CHE, has been increasing with the rapid increase in demand for health care and universal medical insurance coverage. In China, the number of diagnosis and treatment visits sharply jumped from 6.89 billion in 2012 to 7.93 billion in 2016, and the number of inpatients rose from 0.18 billion in 2012 to 0.23 billion in 2016. Thirdly, a study comparing 14 countries discovered that higher CHE incidence was significantly related to higher OOP health expenditures [34], and the financial protection level of medical insurances remained low in China, resulting in higher OOP health expenditures and incidence of CHE. Lastly, as China’s population is aging quickly, and increasing numbers of older adults suffer from multiple health problems, leading to the rapid escalation of health expenditures, thus increasing the incidence of CHE.

We also examined the equality status of CHE using concentration index. It is also worth noting that concentration indices of CHE were all negative from 2012 to 2016, indicating a concentration of CHE occurrence among the poorer households. This finding was concordant with Xu et al.’s study, which indicated that households with low economic status had a higher probability of incurring CHE than the rich in Shaanxi rural areas [12]. As mentioned above, the equality status of CHE even got worse over these years. This finding was also consistent with the result of Gu et al. [35], who found that income-related inequality of CHE occurrence was somewhat aggravated between 2009 and 2010 in the rural areas of Jiangsu Province.

Another important finding in our study is that the four medical insurances exhibit significantly different financial protection effects. Such a finding could be attributed to the different insurance policies. It should be noted that households covered by basic health insurance schemes, including UEBMI, URBMI, and NRCMI, demonstrated higher headcount, overshoot, and MPO. This finding is consistent with the finding of Yang et al. [23], in which households insured by basic health insurance schemes demonstrated higher headcount and overshoot. Additionally, the random effects panel Probit regression results also show that residents covered by basic health insurance schemes could not decrease the odds of CHE occurrence. This was consistent with the finding of Wang and Xu, who reported that basic health insurance schemes could not reduce the risk of CHE and may even increase it using China Health and Retirement Longitudinal Study (CHARLS) database [36]. Although China has achieved the objective of universal insurance coverage, the actual reimbursement ratio and range of basic health insurance schemes remain at a relatively lower level due to the restriction of financing, thus the protection effects are quite poor. In contrast, the headcount, overshoot, and MPO of residents insured by SMI were obviously lower than other medical insurances in 2016, indicating that the protection effect was much better. The reason may be due to the fact that SMI has higher reimbursement level and more flexible insurance product design. Moreover, decreasing overshoot and MPO of households insured by SMI indicate that CHE intensity became relatively lower. SMI is an important part in the current multiple level medical insurance system, and plays a significant role in protecting against CHE, whilst its proportion in the medical insurance system is too small, leading to the fact that SMI cannot meet residents’ needs for higher level insurance and basic medical insurance schemes overburden. Similar to the reports from previous studies, our study revealed that individual characteristics, such as being unmarried, unhealthy, and having chronic diseases, may increase the likelihood of experiencing CHE. This finding will be useful in identifying social groups vulnerable to CHE occurrence. Lastly, regression results by gender show that male residents were better protected by SMI than female residents. The main reason may lie in that male residents are better at utilizing SMI than female residents. Moreover, the regression results by health status reveals that SMI protected healthy residents from experiencing CHE, while it did not efficiently protect unhealthy residents from CHE incurrence, indicating that its financial protection equality status should be further improved.

On the basis of the findings of this study, medical insurance should be improved in several aspects to protect from CHE. Firstly, medical insurance policy should be more equity-oriented to achieve its policy goal, and it is rather necessary to promote the integration of medical insurance systems between urban and rural areas, and decrease benefit differences to improve the financial protection equality [37, 38]. Secondly, it is of policy importance to constantly increase government spending on health care services and enlarge the pooling of medical insurances, and reasonably improve the financing level of NRCMI to enhance the reimbursement ratio and range, thereby improving financial protection level and relieving the economic burdens of diseases on households [39]. Thirdly, it is feasible to promote economic development, expand employment, and increase household income to efficiently improve the ability to prevent catastrophic health spending [40]. On the other side of the coin, considering the fact that health expenditure has been increasing sharply over the years, increased attention should be paid to reforming health care services payment, such as applying diagnosis-related groups (DRGs) to medical insurance prepayment more widely, to strengthen the control of escalating health expenditure [41, 42]. Fourthly, China ought to further develop SMI utilizing market mechanism in order to provide residents with higher level insurance services and decrease the burden of basic medical insurance schemes in the present circumstances.

This study does have several limitations, which should be noted in future research. Firstly, the panel data used in this study were self-reported, and recall bias may exist because of a long recall period, which might affect the accuracy of results to a certain degree. In addition, the self-reported health status in this study may be significantly impacted by health-consciousness level of respondents. Secondly, SMI could not be subdivided into the five specific types of medical insurances mentioned above due to the unavailability of data, which limited the depth of analysis in this study. Thirdly, OOP health expenditure used in this study did not include indirect health care service expenditures, such as transportation expenses, accommodation expenses, and lost income caused by illness, which may understate the financial consequence of health expenditure, thereby leading to an underestimation of the incidence of CHE.

Despite these limitations, this study provides useful information on the development of medical insurance in China, and identifies specific areas where further study is needed. To the best of our knowledge, this study is the first to investigate the relationship between medical insurance and CHE occurrence and compare the financial protection disparities of different medical insurances using panel data. Panel data can reflect changes in the characteristics of variables and correctly explain the relationship between them [43]. Given the high values of the effect of medical insurance on CHE, our findings are particularly important for health policy makers to identify solutions aimed at improving medical insurance in China. In addition, this study provided relevant evidence from China and added to the growing body of international literature on the impact of medical insurance on CHE.

## Conclusion

In summary, the results of this study suggest that CHE incidence and intensity became relatively higher among households. In addition, CHE occurrence was concentrated among the poorer households and the equality status worsened. Moreover, financial protection effects of the four medical insurance schemes against CHE varied significantly. Specifically speaking, SMI could reduce the odds of CHE occurrence, while basic health insurance schemes could not. Furthermore, the protection effect of SMI against CHE shows significant gender and health status disparities. Further reform is required to reduce the disparities of reimbursement level for different medical insurances by promoting the integration of urban and rural medical insurance systems.

## Data Availability

The data used in this study were derived from China Family Panel Studies in the years of 2012, 2014, and 2016. Available at: http://www.isss.pku.edu.cn/cfps/. Accessed 20 Aug 2018.

## Abbreviations

CFPS: China Family Panel Studies
CHE: Catastrophic health expenditure
OOP: Out-of-pocket
MPO: Mean positive overshoot
CI: Concentration index
UEBMI: Urban Employee Basic Medical Insurance
URBMI: Urban Resident Basic Medical Insurance
NRCMI: New Rural Cooperative Medical Insurance
SMI: Supplementary Medical Insurance
